# Hierarchical ZnO Nanostructures with Blooming Flowers Driven by Screw Dislocations

**DOI:** 10.1038/srep08226

**Published:** 2015-02-04

**Authors:** Chengzi Huang, Run Shi, Abbas Amini, Zefei Wu, Shuigang Xu, Linfei Zhang, Wei Cao, Jiangwei Feng, Haisheng Song, Yantao Shi, Ning Wang, Chun Cheng

**Affiliations:** 1 Department of Materials Science and Engineering and Shenzhen Key Laboratory of Nanoimprint Technology, South University of Science and Technology, Shenzhen, 518055, China; 2 School of Computing, Engineering and Mathematics, University of Western Sydney, Kingswood, NSW 2751, Australia; 3 Department of Physics, Hong Kong University of Science and Technology, Hong Kong, China; 4 Wuhan National Laboratory for Optoelectronics and the School of Optical and Electronic Information, Huazhong University of Science and Technology, Wuhan 430074, China; 5 State Key Laboratory of Fine Chemicals, School of Chemistry, Dalian University of Technology, Dalian 116024, China

## Abstract

Hierarchical ZnO nanostructures with a large yield were fabricated by a simple thermal evaporation method. For the first time, novel ZnO flowers were observed blooming at certain sites of a variety of spines, identified as Zn-terminated polar (0001) planes or tips. The spines for as-synthesized hierarchical structures can be nanowires, nanobelts, nanodendrites, nanobrushes, etc. This growth phenomenon determines the key role of polar sites in the fabrication of hierarchical structures. The spiral feature of ZnO flowers indicates an unusual screw dislocation driven growth mechanism, which is attributed to a high concentration of Zn vapor.

Recent years, one-dimensional (1D) or quasi-1D ZnO nanostructured materials have received special attention due to their unique properties and numerous potential applications. High chemical stability, low threshold intensity, wide band gap of 3.37 eV, and a large exciton binding energy of 60 meV make ZnO an excellent candidate for the fabrication of electronic and optoelectronic nanodevices^1^. To date, a variety of nano-sized ZnO with different morphologies have been synthesized, such as nanowires^2^,^3^, nanobelts^4^, nanocombs^5^, nanosprings^6^, tetrapodlike nanostructures^3^,^7^, nanotubes^8^, nanonails^9^, and nanohelices^10^. The diversity of the ZnO crystal morphology makes it suitable for multifunctional applications in electronics, photonics, and even bioelectronics technologies^1^,^11^. As such, a fundamental understanding of the controlling parameters in ZnO crystal growth can further empower the development of novel functional devices.

Generally, ZnO favors a wurtzite lattice structure which has two important inherent characteristics: a) the presence of polar surfaces such as {0001} resulting from Zn- or O- terminated atomic planes, and b) abundant symmetrical structures with two major 6-, 2-fold symmetries and their sub symmetries. Extensive experimental efforts have proven that these two characteristics make ZnO possess of incomparable flexibility in designing novel and complicated hierarchical nanostructures^12^,^13^,^14^,^15^,^16^. To date, a lot of branch morphologies such as nanorods, nanoribbons^14^, nanonails, nanoplates^15^, lotiform-like nanostructures, *etc*.^16^ have been reported. These branches can be totally attributed to the growth parallel or perpendicular to the [0001] direction while the flower-like branches with petals grow from other directions were rarely described. Herein, this paper reports for the first time, that the high-yielding synthesis of hierarchical structures of ZnO flowers bloomed on several kinds of spines. The process of formation and the mechanism, of the hierarchical structures, were investigated on the basis of structural information provided by electron microscopy analysis, and morphology analysis utilizing the law of constancy of interfacial angles.

## Results

**Figure 1:**
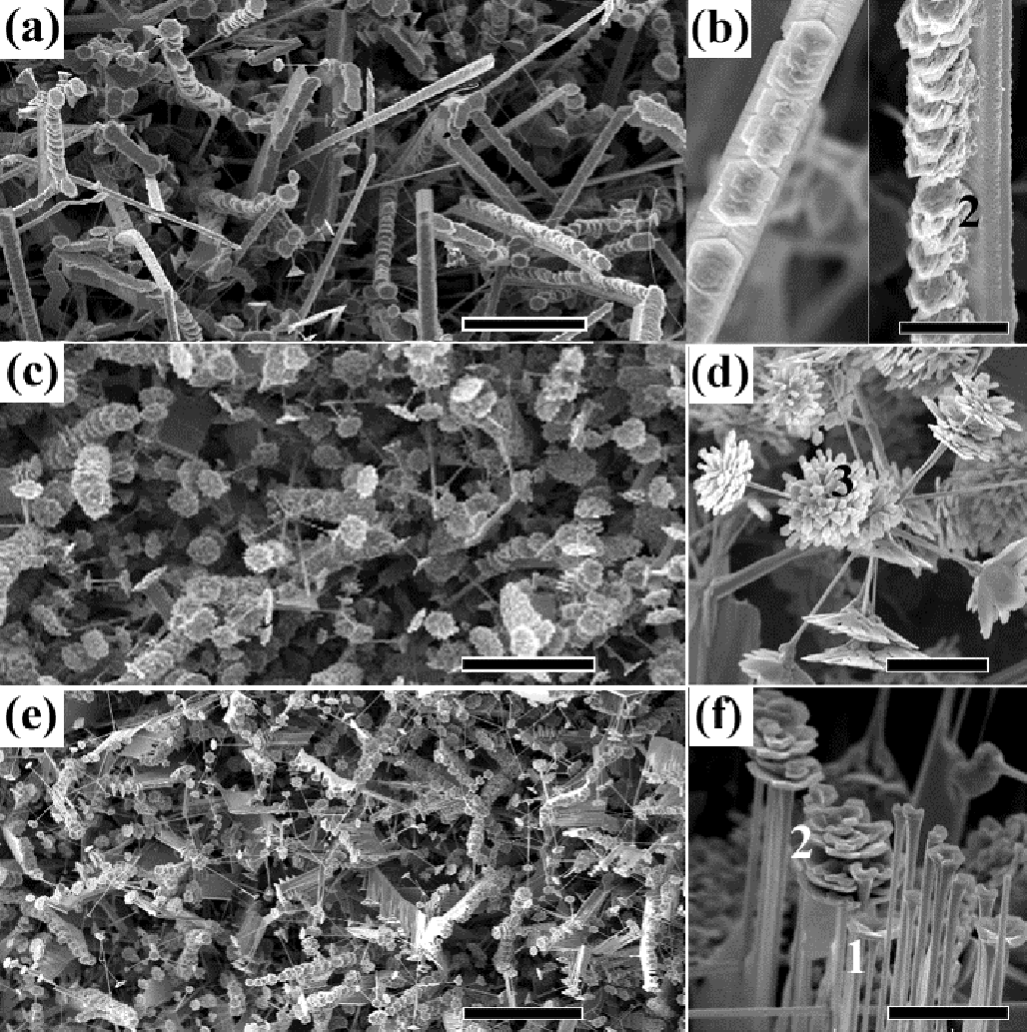
SEM images of ZnO hierarchical structures as-synthesized. (a), (c) and (e) are typical morphologies from the low temperature region to the high temperature region. (b), (d) and (f) are the corresponding enlarged images of (a), (c) and (e). The left panel in (b) is a type IA hierarchical structure with the spine of a V-shape belt and the right panel is type IB hierarchical structure with the spine of a rectangular belt. Numbers 1, 2 and 3 in (b), (d), and (f) mark single-layered, multilayered and multifid flowers, respectively. The scale bars are 100 μm for (a), (c) and (e), 20 μm for (b) and (d), and 10 μm for (f).

Scanning electron microscopy studies in Fig. 1 show three dominant morphologies with large yield from low temperature deposition region to high temperature deposition region: belts (type I, 900~1025°C, Fig. 1a, b), dendrites (type II,1025~1150°C, Fig. 1c, d), and brushes (type III,1150~1175°C, Fig. 1e, f), all with flower-like growth at specific sites. Though the observed hierarchical morphologies are complex and multifarious, they are made up of two parts: the spines and the branched flowers.

**Figure 2:**
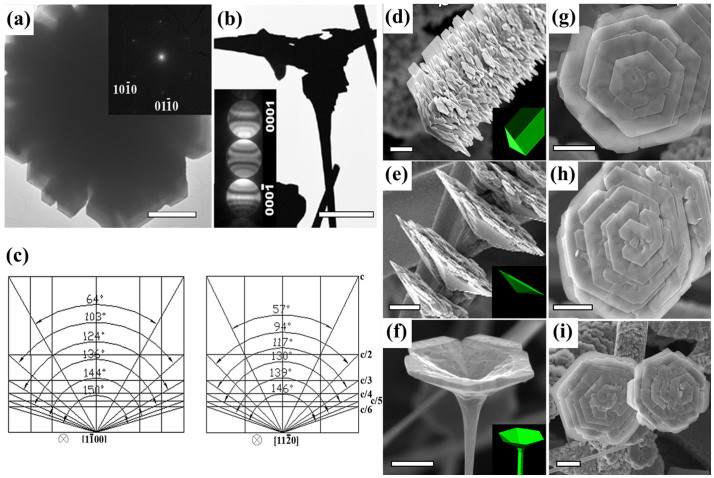
(a) TEM image of a nanoflower with the beam direction along the open direction of its flat surface. The inset is the SAED pattern of the nanoflower which determines the nanoflower open direction toward [0001] and the single crystal structure of the flower. (b) TEM image of a nanoflower with the beam direction perpendicular to the stem. The inset is the CBED pattern of (b) taken along 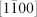 showing that the nanoflower grew from the Zn-terminated polar (0001) site. (c) the projection maps of nanoflowers that consist of different 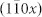 (x = 1,2,3,4,5,6) viewed along the 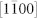 and 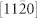
directions. (d) and (e) are two typical nanoflowers with large opening angles. (f) is a typical nanoflower with small opening angle. The insets in (d) – (f) are the corresponding three dimensional crystal models. (d) and (e) consist of 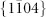 planes and (f) consists of 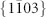 planes. (g) Nanoflowers with left handedness, (h) right handedness and (i) two screws with a similar handedness. The scale bars are 10 μm for (d) and (e), 5 μm for (b) and (g) – (i), and 2 μm for (a) and (f).

Figure 2a shows a low-magnification TEM image of a typical flower with the open direction parallel to the electron beam. The outer sides of the flower show regular corners of 120°, indicating a crystalline structure. The selected electron diffraction (SAED) pattern inserted in Fig. 2a was taken from the whole flower. The SAED pattern is indexed to be hexagonal wurtzite ZnO with zone axis [0001]. The diffraction spots are clear and round and no additional points are present, indicating that the whole flower is a single crystal without stacking faults. Further, the electron beam was focused on the edge of the flower in Fig. 2b for the convergent beam electron diffraction (CBED) analysis to study the polar surface orientation of ZnO flowers (for a detailed description of the method of the CBED experiments please refer to references 14, 17). The experimental CBED result in the inset of Fig. 2b indicates that the open direction of the flower is parallel to the Zn-terminated [0001] direction.

Because the ZnO flowers presented here are single crystalline, crystallogeometry analysis can be used based on the exterior morphologies of a perfect single crystal obeying the law of constancy of interfacial angles^18^. Three dimensional crystal models were constructed to identify the petal planes. Fig. 2c gives the projection maps for the models that consist of different planes of 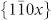 (x = 1, 2, 3, 4, 5, 6) viewed along the 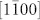 and 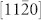
directions. It is noted that the opening angles of these flowers are sensitive to the deposition temperature: the flowers at low temperature deposition region (Figs. 1a and b, Figs. 2d and e) have larger opening angles than those at higher temperature deposition region (Figs. 1c–f, Fig. 2f). By comparing the crystal models with the observed flower crystals, we found that the model consisting of 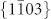 planes matches well with the flowers with large opening angles (Figs. 2d, e and their insets), and the model consisting of 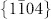 planes matches well with those with small opening angles (Figs. 2f and its inset). As demonstrated in Fig. 1, these ZnO flowers come into bloom on spines just like the flowers in nature and they can be single-layered, multilayered and multifid (marked with number 1, 2 and 3 respectively in Figs. 1b, d and f).The SEM observation of the developing branches of the flowers reveals a spiral growth. As such, the final morphologies of flowers with large opening angles always show a distinct trend towards handedness (Figs. 2g–i). After analysis of nearly one hundred different flowers with a random selection over chirality, it was found that about 90% are right-handed (Fig. 2h) and only less than 10% are left-handed (Fig. 2g). A small portion of flowers with two screws in the same rotation direction are also observed (Fig. 2i).

**Figure 3:**
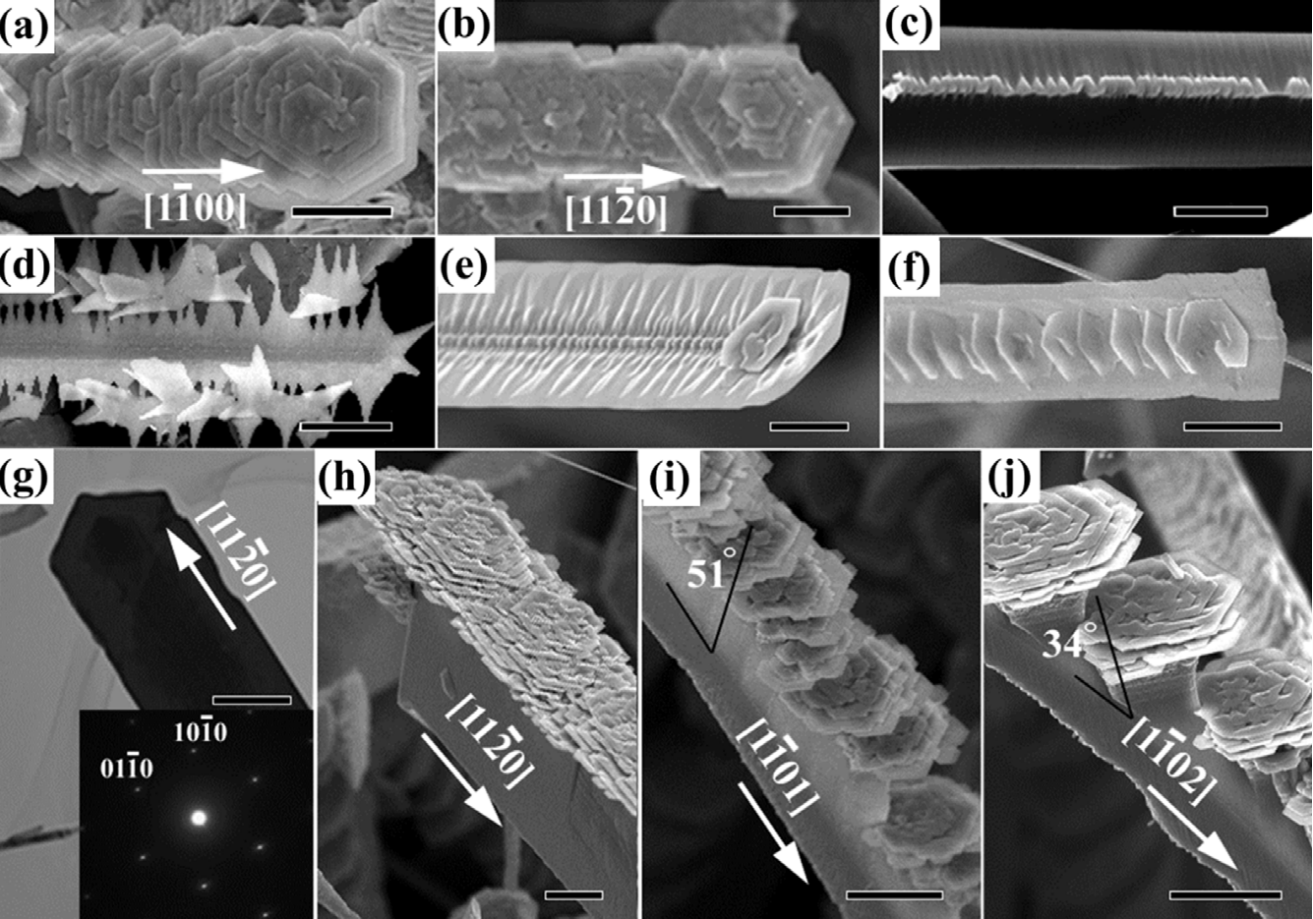
SEM images of (a) Belt-like hierarchical structures along 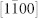, (b) along 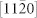
with flowers opening toward the [0001] direction. (c) Some particles are observed on the ridge of the 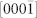 side of the belt-like hierarchical structure. (d) – (e) Developing morphologies of belt-like hierarchical structures. (f) TEM image and SAED showing that the belt is a single crystal with the spine along 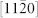
and its projection plane perpendicular to [0001]. (g) – (j) are SEM images of flowers grown on thick rectangular belts along the 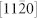, 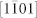 and 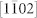 directions. The scale bars are 10 μm for (a), (b), (d) and (h) – (j), 5 μm for (e), 2 μm for (c) and (f), and 200 nm for (i).

**Figure 4:**
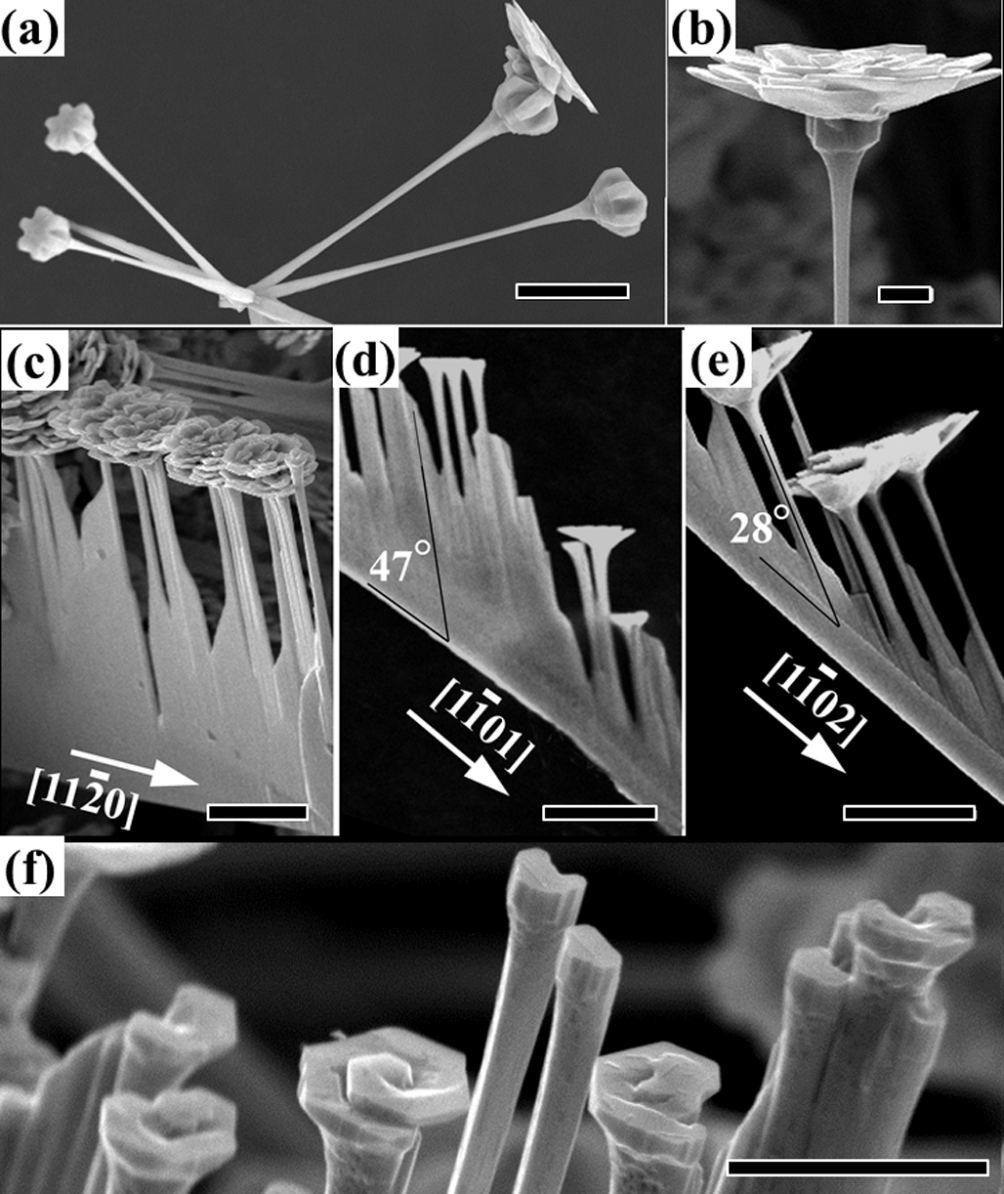
(a) SEM image of developing morphologies of dendrites with flowers grown at the frontier tips that appearing in a six-symmetrical ball form. (b) SEM image of a flower grown on a ball. (c) – (e) SEM images of flowers grown on thick rectangular belts along the 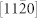, 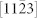 and 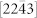 directions. f) Flower growth initiating from screw dislocations. The scale bars are 10 μm for (a) and (c) – (e), 5 μm for (f), and 2 μm for (b).

As shown in Fig. 1b, there are two kinds of type I - belt-like hierarchical structures coexisting in the low deposition temperature region: Type IA has spines of V-shape belts (see the left panel in Fig. 1b, Figs. 3a–g) and type IB has spines of rectangular belts (see the right panel in Fig. 1b, Figs. 4h–j). The flowers of type IA and IB are assembled in-line only on one side of these belts. Fig. 3c and Figs. 3h–j depict the other sides of these belts which are smooth apart from nanoparticles growth along their axes. This phenomenon is produced by different growth rates on the active (0001)-Zn and inert 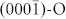 terminated surfaces^12^. ZnO hexagonal flowers have two oriented stands on the spines in type IA, that is, either parallel or perpendicular to the axis of the spines (Figs. 3a and b). This indicates the existence of ZnO spines with two usual growth directions: 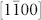 and 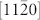.Our observation shows that the former stand orientation exists much more than the latter. In addition, Figs. 3d–f show the intermediate structures toward the formation of type IA hierarchical structures. It is clear that the extended growth of triangle thin films alongside the one-dimensional spine (Fig. 3d) and their integration lead to the formation of the long V-shape belt (Fig. 3e). It was found that small isolated islands appear on the axis (Fig. 3e) and flowers originate from these islands by helical growth (see Fig. 3f). These petals of flowers extend in the same direction of the wings. The TEM image of a type IA hierarchical structure with the hexagonal side parallel to the axis of spines is shown in Fig. 3i and the corresponding SAED in the inset of this Figure identifies the growth direction of the spine is along 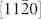. It was found that the color in the middle part of the V-shape belt is much darker comparing to that in its edge. This is attributed to faster growth in the middle part because the naked Zn-terminated sites are quite active, supporting a self-catalytic growth^2^. Figs. 3h–j show typical SEM images of type IB hierarchical structure with flowers grown on thick rectangular belts. Spines of belt with three main growth orientations are the main products and the flowers fabricated on these spines have either perpendicular or inclined open directions the same as the spine direction. Comparing these results with Fig. 2c, the growth directions of spines are determined as 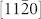, 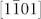 and 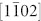. And all the flowers and thin films that extend from the spines in type I have large opening angles consisting of 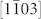 planes (Figs. 2d and e).

Figure 1c shows a type II hierarchical structure with flowers blooming at the tips of dendrites. Each branch consists of a ball and a flower with multilayered petals (Fig. 4a). Six-folded symmetrical balls are always found at the frontier end of the stems. The flowers grown on these balls (Figs. 4b–d) have small opening angles and consist of 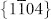 planes, identified by comparing the flowers with the models in Fig. 2f and plane projections in Fig. 2c. Figs. 1e and f show the typical type III hierarchical structures, with flower branches grown on brushes. These flowers always consist of multilayered petals. Figs. 4c–e show the brushes with three main growth orientations of 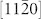, 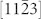 and 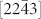. Fig. 4f presents some developed branches of flower where helical growth circling of [0001] stem is clearly observed. These flowers have small opening angles consisting of 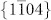 planes.

## Discussion

As pointed out, the formation of hierarchical nanostructures generally can be divided into two major stages^12^,^13^,^14^,^15^,^16^,^19^. The first stage is a fast growth of the spines with different morphologies (nanowires, nanobelts and more complex structures like dendrites) depending on deposition temperatures. These different spines grow with naked Zn-terminated (0001) planes on their heads or sides as identified by the CBED analysis (inset in Fig. 2b). It is known that the Zn-terminated surface is catalytically active and thus induces secondary growth whereas the O-terminated surface is inert^14^. Furthermore, the closer to the source, the higher the temperature and, therefore, the denser the Zn vapor and O_2_ vapor from the decomposition of ZnO at 1300°C. As a result, in the second stage, nanostructures near the source convert into nanobrushes or wide belts through a secondary fast growth on naked (0001) surfaces^12^,^13^,^14^,^15^,^16^. In addition, the surface deposition of the source atoms contributes to the relatively slow growth on other surfaces leading to the thickening of nanostructures. Different from previous work on the growth of ZnO hierarchical nanostructures, ours has an additional process for the flower growth besides above two growth stages due to a prolonged growth time of 2 hours. The growth in the third stage is rather different from that at the second stage: flowers grow from the screw dislocations at naked (0001) sites of the spines formed in advance. It is worth mentioning that a large amount of grey powder was found deposited at the low deposition temperature region of ~300–400°C. This grey powder was identified as irregular thin Zn nanowires by TEM. However, when decreasing the reaction time to one hour, this grey powder did not exist and only the products of the second stage (nanowires, nanobelts and nanobrushes) were observed. Based on these experimental results, we believe that the flower growth is related to the fact that Zn vapor is enriched in the low temperature region in the third stage. Apparently, in order to reach the third growth stage, adequate amount of ZnO powder (20g in experiments) is required, otherwise no ZnO flowers can be observed.

In most work reported for the growth of ZnO nanostructures by the thermal evaporation method, the growth time was always limited less than 1 hour. With the high temperature above 1200°C and pressure less than 0.1 torr, ZnO can be easily decomposed into Zn vapor and O_2_. Some Zn vapor reacted with O_2 _to form ZnO crystals in the deposition regions. The remaining Zn vapor was carried downstream and condensed in the low temperature region with redundant O_2_ being pumped out continuously. Therefore, at the beginning of thermal evaporation, the concentration of Zn vapor in the high temperature region was much larger than that in the low temperature region. However, as long as the reaction time is long enough (as to our case, the time is larger than 1 hour according to experiment results), there will be a dramatic change in the distribution of Zn vapor concentration from the ZnO powder source to the low temperature region. That is, Zn vapor continuously accumulated in the low temperature region and finally reached an extremely high density, which is supported by the observation of gray Zn powder in the low temperature region. Previous studies^8^,^13^ have shown that the fastest growth along the [0001] direction of ZnO nanostructures transits to the one perpendicular to it in the presence of a high concentration of Zn vapor. As a consequence, ZnO nanostructures show pin-like morphologies. Similarly, in the present case, the special ZnO flower-like morphologies are attributed to the suppressed growth along the [0001] direction and fast growth along new directions, such as 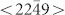 and 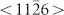 that those petals stretched out along (Figs. 2d–f). Because of an extremely high Zn vapor concentration in the low temperature region after a long time thermal evaporation, the growth speed along [0001] was hindered further. Consequently, flowers in the low temperature region have larger opening angles (Fig. 2f) while, in the high temperature region, smaller opening angles are expected (Figs. 2d and e). The SEM observation shows that, due to the much higher Zn vapor concentration, the tips of these flowers developed to flat surfaces near the deposition region of Zn powder while, for nanobrushes near the ZnO source, few flowers were found due to the relatively low Zn vapor concentration.

The growth of ZnO nanostructures synthesized by the thermal evaporation methods without introducing metal-catalysts has always been regarded to follow a self-catalytic Vapor-Liquid-Solid (VLS) mechanism^2^. So does the growth of our ZnO hierarchical nanostructures in the first two growth stages while it changes to an unusual screw dislocation driven growth for ZnO flowers in the third growth stage. The spiral feature at the center of the ZnO flowers is obvious due to the steps generated by a screw dislocation (Fig. 4f). It has been frequently observed that screw dislocations are associated with growth of crystal in the micrometer-sized dendrite or whisker geometries. However, in nanowires, no screw dislocations had been evidenced until recently J. Song *et al.* fabricated hierarchical pine tree PbS nanowires with helically rotating branches via chemical vapor deposition (CVD) reactions^20^. They also demonstrated the screw-dislocation-driven growth of ZnO nanowires and nanotubes via solution-phase methods by introducing screw dislocations from epitaxial substrates or applying low supersaturation conditions^8^. To our knowledge, the growth of ZnO flowers here is the first demonstration of controllable ZnO nanostructure growth driven by screw dislocations via the thermal evaporation approach. Undoubtedly, the as-grown flower-decorated hierarchical nanostructures largely enrich the morphology library of ZnO crystals. Most importantly, the finding that the screw dislocation driven growth can be triggered by high concentration of Zn vapor may enable us the ability to “engineer” the dislocation sites for the tailoring of ZnO nanostructures with controlled location and morphology suited for specific purposes.

## Conclusions

In conclusion, we reported the growth of a new group of ZnO hierarchical nanostructures with flowers blooming at polar sites of various spines using a simple thermal evaporation method. It is shown that Zn-terminated polar planes play a key role in the fabrication of hierarchical structures. The unusual Zn vapor concentration distribution is proposed as the cause for the screw dislocation driven ZnO flower growth. Our strategy of controlled growth of ZnO hierarchical nanostructures by the combination of polar sites and dislocations promisingly inspires a new way to tailor ZnO nanostructures for the design of novel functional devices that can be applied in solar cells^21^, nanogenerators^22^, and sensors^23^.

## Methods

Synthesis of these hierarchical structures was carried out by using a simple vapor deposition process. Commercially available ZnO powder was placed in the center of a horizontal tube furnace. Three pieces of polycrystalline alumina substrates were placed downstream in the lower temperature region of a horizontal tube furnace. The furnace was heated to 1300°C and kept for 2 hours at a pressure of 2 × 10^−2^ Torr. After the growth, the furnace was gradually cooled down to room temperature. In addition to the white products collected from the substrates, some gray powder was also sampled at the 350°C-temperature zone. The X-ray diffraction (XRD) data confirmed that the as-synthesized white sample was wurtzite ZnO. The morphologies and structures of these white samples were analyzed by a scanning electron microscopy (SEM) and a transmission electron microscope (TEM). Energy dispersive spectrometry (EDS) identified only Zn and O with a ratio of ~1:1 existed representing no impurities in the as-synthesized products. The optical observation revealed that the final product appeared white and covered the three alumina deposition substrates with a high yield.

## Author Contributions

C.C. designed the research. C.C., R.S., C.H., Z.W., S.X., L.Z., W.C., J.F. performed the experiments. A.A., H.S., Y.S., N.W. incorporated in the interpretation of experimental results. All authors reviewed the manuscript.
